# Whole-brain in situ postmortem MR imaging using a combination of sequences for cortical lesion detection in multiple sclerosis

**DOI:** 10.1007/s11547-026-02185-1

**Published:** 2026-02-12

**Authors:** Piet M. Bouman, Jeroen J. G. Geurts, Laura E. Jonkman, Menno M. Schoonheim, Frederik Barkhof, Lukas Haider

**Affiliations:** 1 MS Center Amsterdam, Vrije Universiteit Amsterdam, Amsterdam Neuroscience, Amsterdam UMC VUmc, De Boelelaan 1117, 1081HV Amsterdam, The Netherlands; 2 Anatomy and Neurosciences, Amsterdam UMC Location Vrije Universiteit Amsterdam, De Boelelaan 1117, Amsterdam, The Netherlands; 3 Amsterdam Neuroscience, Brain Imaging and Neurodegeneration, De Boelelaan 1117, Amsterdam, The Netherlands; 4 Radiology and Nuclear Medicine, Amsterdam UMC Location Vrije Universiteit Amsterdam, De Boelelaan 1117, Amsterdam, The Netherlands; 5 NMR Research Unit, Queen Square Multiple Sclerosis Centre, Queen Square Institute of Neurology, University College London, London, UK; 6 Department of Biomedical Imaging and Image Guided Therapy, Division of Neuroradiology and Musculoskeletal Radiology, Medical University Vienna, Vienna, Austria

**Keywords:** Multiple sclerosis, Cortical lesions, Magnetic resonance imaging, Diagnosis

## Abstract

**Purpose:**

Cortical lesions are specific for multiple sclerosis but remain challenging to detect using magnetic resonance imaging (MRI). While numerous MR sequences have been evaluated individually, their combined performance in clinical routine settings has not been validated histopathologically. This study aimed to determine the detection rate of histopathologically validated cortical lesions using combined assessment of multiple MRI sequences in postmortem in situ imaging.

**Material and methods:**

Five MRI sequences [phase-sensitive inversion recovery (PSIR), double inversion recovery (DIR), fluid-attenuated inversion recovery (FLAIR), 3D-T₁, and proton density (PD)/T₂] were acquired at 3 T for 18 patients with multiple sclerosis using postmortem in situ whole-brain imaging. A total of 66 tissue samples were collected and stained for myelin to identify cortical lesions types I–IV. Cortical lesions were assessed prospectively on MRI (blinded to histopathology) and retrospectively (with histopathological knowledge) using combined sequence evaluation and consensus reading.

**Results:**

Histopathological analysis revealed 115 cortical lesions in 16/18 patients (4 type I, 43 type II, 61 type III, 7 type IV). Prospective assessment using all MRI sequences combined detected 20/115 (17.4%) cortical lesions with 100% specificity. The combination of DIR and PSIR sequences showed a 43% relative increase in detection compared to conventional sequences. Retrospective assessment with histopathological knowledge increased detection to 46/115 (40.0%) lesions, with DIR and PSIR in  combination providing an 18% relative improvement.

**Conclusion:**

Despite using advanced MRI sequences in a highly controlled postmortem setting, cortical lesion detection remains limited at 17.4%. The combination of DIR and PSIR sequences provides the most effective approach, significantly outperforming conventional sequences. These findings establish a reference benchmark for cortical lesion detection rates and highlight persistent limitations of current MRI technology for identifying cortical pathology in multiple sclerosis.

## Introduction

Multiple sclerosis is an inflammatory, demyelinating and neurodegenerative disease of the central nervous system [1, 2]. While grey matter involvement in MS has been reported early on in science, its extent and clinical relevance emerged only with the advances of immuno-histochemistry [3–7]. More specifically, cortical lesions were found to be present in the early stages of multiple sclerosis, highly specific for the disease, have a high prognostic value for the disease course, and to be associated with physical and cognitive decline [8–14].

Visualisation of cortical lesions has fuelled a lot of debate, in terms of preferred sequences and criteria to identify lesions [15–20]. Several works have performed head-to-head comparisons between different MRI sequences: both clinical sequences such as T_1_, T_2_, and fluid-attenuated inversion recovery (FLAIR), and more advanced sequences such as double inversion recovery (DIR) and phase-sensitive inversion recovery (PSIR) have been assessed in relation to histopathological ground truth [21–23]. A recurring finding in those endeavours is the staggering discrepancy between the small number of lesions that are discernible on the MRI and the amount of lesions that are present in the tissue [24, 25].

Thenceforth, a plethora of studies has attempted to bridge this gap, through development of new MRI sequences, quantitative MRI, or increasing magnetic field strength [26–31]. There is a high variation in the reported lesion frequencies detected using MRI and study protocols often do not match the clinical routine for cortical lesion identification, which is seldom based on single MR sequences, but rather an interpretation based on an impression derived from all available images combined.

Many prior studies assessing cortical MRI lesion sensitivity are performed with ex vivo brain slices, assessing formalin-fixed tissue which alters contrast, and are mostly sequence-to-sequence comparisons. The combined performance of routinely applied 3D pulse sequences, as used in clinical settings, has not been validated histopathologically.

In this work, we aimed to determine the number of histopathologically validated cortical lesions that can be detected on postmortem in situ imaging 3 T MRI. Thus, all available sequences were assessed in combination and blinded to histopathology, followed by unblinded histopathological validation. Our data provide a reference benchmark of cortical lesion detection rates for a high-quality clinical image setting.

## Methods

### Patients and autopsy procedure

Imaging data and tissue samples were obtained following the standardised rapid autopsy protocol of the Amsterdam MS Center and the Netherlands Brain Bank [32]. All patients for whom both in situ MR imaging and histopathological data that were available were included for the study. All patients provided written informed consent during life for brain autopsy, imaging, tissue storage, use of tissue and forthcoming images, and use of their medical records for research purposes, in accordance with the Netherlands Brain Bank prior to death. Permission for the autopsy protocol was granted by the Amsterdam UMC institutional ethics review board, approval number 2009/148.

### MRI

Postmortem in situ (brain in cranium; people with multiple sclerosis often have not been scanned for a long period prior to death) imaging was performed on a 3 T whole-body scanner (GE Signa HDxt, Milwaukee, WI, USA), using an eight-channel phased-array head coil. The imaging protocol included a 3D-T_1_-weighted fast-spoiled gradient echo sequence (FSPGR; TR 6.66 ms, TE 2.93 ms, TI 450 ms, 15° flip angle, sagittal 1.0 mm slices, 1.0 × 1.0 mm^2^ in-plane resolution, TA 5 m 7 s), a 2D axial dual-echo proton density sequence (PD)/T_2_-weighted TR 4246 ms, TE 20/112 ms, axial 3.0 mm slices, 1.0 × 1.0 mm^2^ in-plane resolution, TA 4 m 41 s), a 3D-FLAIR sequence (TR 8000 ms, TE 125.9 ms, TI 2147 ms, sagittal 0.97 × 0.97 mm^2^ in-plane resolution, TA 5 m 39 s), a 3D-DIR sequence (TR 8000 ms, TE 126.4 ms, TI 725/4500 ms, sagittal 1.2 mm slices, 0.97 × 0..97 mm^2^ in-plane resolution, TA 9 m 40 s), and a 2D-PSIR (TR 3850 ms, TE 13.1 ms, TI 419 ms, axial 2.0 mm slices, 0.5 × 0.5 mm^2^ in-plane resolution, TA 3 m 20 s).

### Prospective MRI assessment

For each patient, cortical lesions were identified in consensus by PMB and LH in accordance with standard criteria [16]. In case of doubt or disagreement, expert decision was consulted (FB). Since the autopsy protocol consists of tissue sampling based on visible lesions, all regions were assessed for MR-visible cortical lesions, blinded to histopathology, in order to prevent bias. Due to availability and previous literature, MR reading started with PSIR sequences, and all other sequences were simultaneously assessed in parallel to confirm findings and/or assess different imaging planes. On PSIR, cortical lesions were identified as hypointensities compared to surrounding normal-appearing grey matter, involving either part of the cortex or the cortex as a whole [20]. On DIR, cortical lesions were considered hyperintense areas compared to surrounding normal-appearing grey matter, of at least 3 voxels (i.e. 3 mm^2^) in size, in accordance with the guidelines that were developed by the MAGNIMS consortium [16]. For the other consulted sequences, no such guidelines for cortical lesion identification were available. For each identified lesion or enlarged perivascular spaces, the determining sequence was recorded, along with a degree of certainty for each reader. Identified lesions were then classified as *intracortical* (i.e. completely locate inside the cortex, *leukocortical* (i.e. in situated in part in the cortex and in part in the white matter), and *juxtacortical* (i.e. situated in the white matter but touching cortex). Lesions that were completely in the white matter were considered subcortical and not recorded in detail.

### Histopathological staining and tissue assessment

After MRI scanning, the brain was extracted from the cranium and cut into 1-cm-thick coronal slices, according to a standardised protocol. Per-slice tissue sampling was performed, following a standardised protocol added by visual and palpable assessment of the tissue to increase lesion harvest. Tissue samples were formalin-fixated for 48 h and paraffin-embedded (FFPE). These FFPE samples were cut into 10-μm-thick sections. These sections were heated in the steam cooker for 30 min, submerged in Tris–EDTA buffer (10 mM; pH 9.0) for antigen retrieval. Subsequently, endogenous peroxidase was blocked using 1.0% hydrogen peroxide in Tris buffer saline (TBS; pH 7.6). Non-specific binding was blocked through incubation of the sections with 3.0% bovine serum albumin in TBS-tx. Tissue sections were incubated with primary antibody proteolipid protein (PLP; Bio-Rad, Hercules, CA, US) overnight at 4° Celsius. Subsequently, 2-h incubation was performed with biotin-labelled donkey-anti mouse (DoaM; Jackson IgG, Cambridgeshire, UK) 1:400 diluted in TBS-tx. Then, sections were ABC-incubated (Vector, Burlingame, CA, US) and diluted in 1:400 TBS-tx for 1 h. Colour development was performed using 3′3’-diaminobenizidine (DAB) for ten minutes. Last, counterstaining with thionin (Brand, Wertheim, Germany) was performed. A more extended description of the tissue staining protocol is provided elsewhere. [15]

After tissue staining, the stained samples were assessed for presence of cortical lesions, in which four lesion subtypes were distinguished according to pre-defined criteria [33]. Lesions were defined as areas of complete demyelination and classified into one of four types, based on their position in the cortex: mixed grey-white matter lesions (type I), purely intracortical (type II), subpial (type III), and cortex-spanning (type IV) lesions.

### Retrospective MRI assessment

After prospective MRI scoring and histopathological assessment, the included tissue samples were matched to their corresponding regions on MRI, using as many anatomical landmarks (e.g. nearby anatomical structures and morphology of grey/white matter, lesions, etc.) as possible. Then, the prospective scorings were compared to the histopathological presence of lesions, assessing the ratio of lesions that was detected a priori and simultaneously assessing whether a lesion is visible with histopathological feedback at hand (retrospective assessment).

### Analyses

Sensitivity of the MRI sequences was calculated through dividing the number of detected lesions in the prospective and retrospective scoring by the total number of lesions that were present in the histopathology, times 100%. Specificity of the MRI was calculated through division of the total number of lesions that were detected on MRI by the number of histopathologically validated lesions. McNemar's test for paired proportions was used to express added value of DIR to PSIR for cortical lesion assessment. Intraclass correlations coefficients (two-way mixed model with absolute agreement) were used to express reader agreement. Analyses were performed using R Statistical Software (v4.1.2; R Core Team 2021).

## Results

In situ MRI scans and histopathological data were available for a total of 18 patients with progressive multiple sclerosis [mean postmortem delay 4 h 3 min, standard deviation (SD) 1 h 3 min]. An overview of patient demographics is provided in Table 1. In total, 66 tissue samples were available, in which histopathological analysis revealed 115 cortical lesions; 4 were type I, 43 type II, 61 type III, 7 type IV, and 11 white matter lesions. Reliability analysis showed excellent agreement between the readers (ICC 0.92 [95% CI: 0.90–0.94]).

**Table 1:** Demographical data

Patient no	Sex	Age (years)	Postmortem delay (hours:mins)	Disease duration (years)	Disease type	Cause of death
1	F	58	4:00	25	PPMS	Euthanasia
2	F	77	4:00	26	PPMS	Natural
3	F	82	3:40	60	SPMS	Euthanasia
4	F	52	3:40		Unknown	Euthanasia
5	F	75	5:15	25	Unknown	Pneumonia
6	F	65	4:15	16	PPMS	CVA
7	F	39	2:30	8	SPMS	Euthanasia
8	F	77	2:40	27	SPMS	Pulmonary carcinoma
9	F	49	3:55	34	SPMS	Pneumonia
10	F	81	6:20	28	SPMS	Urosepsis
11	F	65	3:30	37	SPMS	Euthanasia
12	M	50	5:30	19	SPMS	Euthanasia
13	M	61	4:50	28	SPMS	Euthanasia
14	F	70	3:52	32	SPMS	Euthanasia
15	M	66	5:00	25	PPMS	Euthanasia
16	M	82	3:00	44	SPMS	Pneumonia
17	F	61	4:30	2	Unknown	Euthanasia
18	F	76	2:30	23	PPMS	Pneumonia

PPMS , primary-progressive multiple sclerosis; SPMS , secondary-progressive multiple sclerosis; CVA , cardiovascular accident. Some of these patients were also included in Bouman et al., [15]

Prospectively, by combining all MR sequence assessments, 20/115 (sensitivity of 17.4%) cortical lesions were detected (Table 2). When only using PSIR as default sequence, 14/115 (sensitivity of 12.2%) cortical lesions were detected. When expanding the assessment to DIR, 6 more cortical lesions (a relative increase in sensitivity of 43%) were identified. Including T_1_, T_2_, and FLAIR sequences to the assessment did not add more lesions to the total. Whereas all but one cortical lesions that were detected on PSIR were also visible on DIR and vice versa, such was not the case for T_1_, T_2_, and FLAIR, on which only a small fraction of cortical lesions were discernible. Table 2 shows the exact numbers of cortical lesions that were detected. All MRI sequences achieved 100% specificity with no false-positive findings, confirming the high accuracy of the consensus reading approach used in this study.

**Table 2:** Prospective scoring results (all lesion types)

	2D-PSIR	3D-DIR*	3D-T_1_	2D-T_2_	3D-FLAIR
Detected	14 (12.2)	20 (17.4)	7 (6.1)	5 (4.3)	10 (8.7)
Undetected	101 (87.8)	95 (82.6)	108 (93.9)	110 (95.7)	105 (91.3)
Total	115	115	115	115	115

Number of cortical lesions that were detected on all sequences, blinded to histopathology. Data are total numbers (percentage/sensitivity). PSIR, phase-sensitive inversion recovery; DIR, double inversion recovery; FLAIR, fluid-attenuated inversion recovery. * Due to the use of PSIR as a reference sequence, the number of lesions that were detected on DIR by definition includes those detected on PSIR

**Fig. 1:**
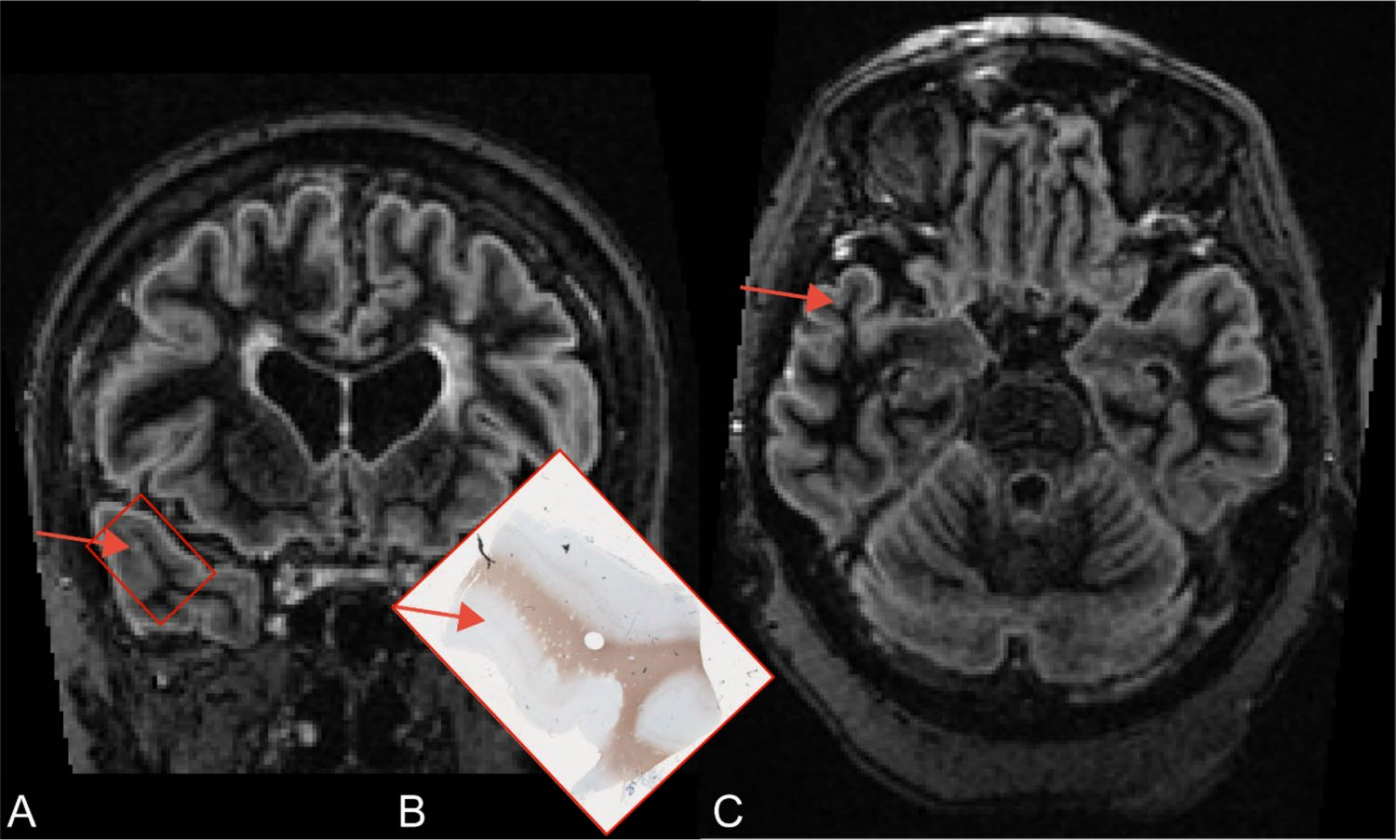
Coronal and axial section of a cortical lesion, in which the lesion is discernible in the coronal but not the axial plane. **A** Coronal double inversion recovery image. **B** Tissue sample showing a type IV cortical lesion indicated by the red arrow, with the histopathological inset in red. **C** Axial double inversion recovery image, in which the lesion location is indicated by the red arrow. Adapted with permission from *Grey Matters Revisited* by P.M. Bouman, 2023

**Fig. 2:**
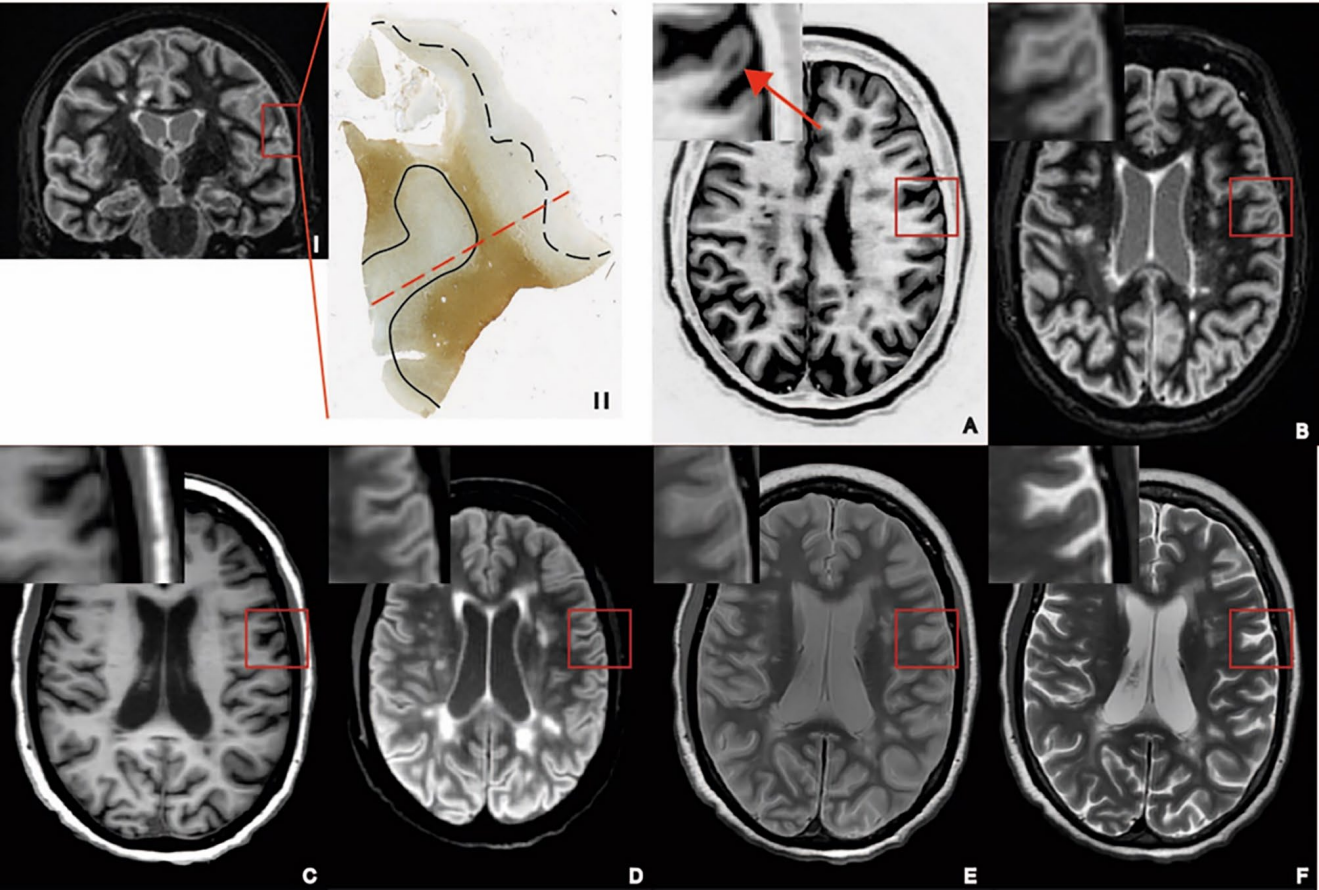
Example of a type IV lesion that is discernible on DIR and PSIR but less to not on other sequences. (I) Coronal double inversion recovery with inset of tissue sample. (II) Tissue sample in which a type IV lesion is demarcated by the black line. The dotted black demarcates a type III lesion that is not discernible on any of the sequences. The red line indicates the level of the axial MRI images in the tissue sample. **A** Phase-sensitive inversion recovery image in which the lesion is discernible. **B** Double inversion recovery image in which the lesion is discernible. **C** 3D-T_1_ weighted. **D** Fluid-attenuated inversion recovery. **E** Proton density. **F** T_2_ weighted

Retrospectively, with knowledge from histological images, 46/115 (sensitivity of 40.0%) lesions were detected, using combined sequence assessment (Table 3). Initially, only assessing PSIR, 39 cortical lesions were detected (sensitivity of 34.0%). When adding DIR, 7 more lesions (total sensitivity of 40%; relative increase in sensitivity of 18%) were detected. There were no lesions visible on T_1_, T_2_, or FLAIR that were not visible on DIR and PSIR sequences. Interestingly, some lesions were visible in coronal but not axial fashion (Fig. 1). The most substantial improvements from prospective to retrospective scoring were observed for the advanced sequences, with 3D-DIR and 2D-PSIR showing increases of 22.8 and 21.7 percentage points, respectively, confirming considerable potential for enhanced detection when histopathological knowledge is available to guide image interpretation (Fig. 2).

**Table 3:** Retrospective scoring results (all lesion types)

	2D-PSIR	3D-DIR*	3D-T_1_	2D-T_2_	3D-FLAIR
Detected	39 (34.0)	46 (40.0)	17 (14.8)	8 (7.0)	11 (9.6)
Undetected	75 (66.0)	69 (60.0)	98 (85.2)	104 (93.0)	104 (93.4)
Total	115	115	115	115	115

Number of cortical lesions that were detected for all sequences, with histopathological feedback available. Data are total numbers (percentage/sensitivity). PSIR, phase-sensitive inversion recovery; DIR, double inversion recovery; FLAIR, fluid-attenuated inversion recovery. * Due to the use of PSIR as a reference sequence, the number of lesions that were detected on DIR by definition includes those detected on PSIR

## Added value of multi-contrast lesion assessment

**Fig. 3:**
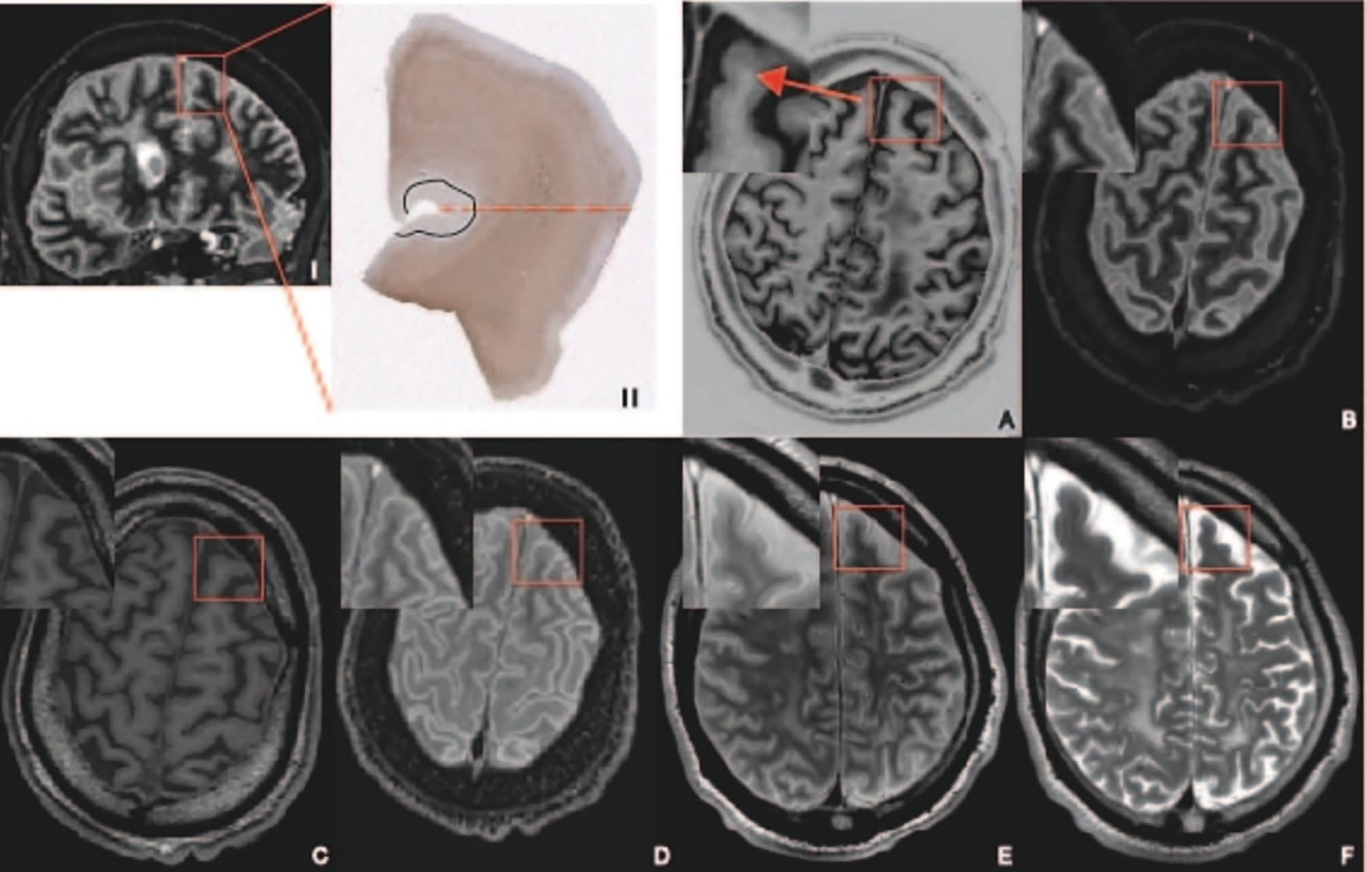
Example of a cortical lesion that is visible in the tissue but not on the MRI. (I) Coronal double inversion recovery image with red inset of tissue sample. (II) Tissue sample with a type III lesion demarcated by the black line. The red dotted line indicates the level of the axial MRI images in the tissue sample. **A** Phase-sensitive inversion recovery. **B** Double inversion recovery. **C** 3D-T_1_ weighted. **D** Fluid-attenuated inversion recovery. **E** Proton density. **F** T_2_ weighted

McNemar’s tests showed that, in the current set-up, DIR demonstrated added value compared to the use of only PSIR for cortical lesion detection in both prospective (χ^2^ = 6.0, *p* = 0.014) and retrospective (χ^2^ = 7.0, *p* = 0.008) assessments (Fig. 3).

## Discussion

In multiple sclerosis, cortical lesions are clinically significant but challenging to detect on MRI. While numerous studies have investigated individual MRI sequences for cortical lesion detection, our study uniquely assessed the combined use of multiple MRI sequences in a setting that closely mimics clinical practice and was histopathologically validated. Using an in situ postmortem approach, we found that the combination of DIR and PSIR sequences increased cortical lesion detection by 43% compared to other sequences. However, even with this optimised multi-sequence approach, only 17.4% of histopathologically validated cortical lesions were prospectively detected. These findings underscore the persistent limitations of MRI in cortical lesion detection, despite using state-of-the-art techniques in a highly controlled setting.

Our results confirm that, despite combining all available sequences from high-quality scans, prospective (20/115) and retrospective (46/115) sensitivity for cortical lesions remains low even in a highly controlled postmortem in situ imaging set-up (with an average postmortem delay of 4 h and without visible artefacts from, for example, blood, loose liquor, or tissue degeneration). Furthermore, the results confirm that detection of cortical lesions depends on the availability of advanced MRI sequences. Having only conventional clinical sequences (T_1_, T_2_, FLAIR) available halves the number of cortical lesions that can be detected. Combining DIR and PSIR leverages the potential of both, as it allows for confirmation of lesions on a sequence with much higher sensitivity than conventional clinical sequences [15, 19]. The results have shown that combined assessment of DIR and PSIR is of added value when compared to PSIR-only assessment. This does not imply that DIR has a higher a priori prospective sensitivity than PSIR, as the sequence was not assessed blinded to its PSIR-counterpart. Former research showed a 5% increase in explained variance when combining DIR and PSIR [15]. The benefit of combining sequences is also expressed in the fact that throughout this work no histopathological false positives were detected. This can be attributed to the consensus lesion identification setting by two readers (experienced with the histopathological appearance of cortical lesions and the included MRI sequences), strictly adhering to the lesion identification criteria. Although this resulted in high specificity, it may also have resulted in slightly lower sensitivity than previously reported [15].

Retrospective MRI sequence assessment (i.e. with histopathological feedback available) showed that, even when combining sequences, purely intracortical lesions (type 2) were never detected prospectively, and subpial (type 3) lesions only to a very limited amount. Thus, results are mostly driven by type IV lesions. Histopathological validation of the data was complicated by the axial fashion in which the images were assessed opposed to the coronal brain sections that were cut in the autopsy protocol [32]. The following translation from axial to coronal led to the finding that not only imaging sequences but also imaging planes contribute to sensitivity: some lesions were visible in coronal but not axial plane as cortex is folded in all directions and 2D sequences inherently produce partial volume artefacts in this region. This may be attributed to the shape and size of the cortical lesions, which are notoriously small. This matter could be taken into account should cortical lesion identification criteria be updated as imaging planes currently do not form part of the criteria and this may further increase specificity. Sensitivity for cortical lesions could be improved by increasing acquisition time or magnetic field strength to 7 T. However, both would come at the cost of clinical feasibility [34]. Sensitivity may also be increased by being less conservative in lesion identification as cortical lesion identification criteria are very strict. To achieve maximum sensitivity for cortical lesions, quantitative MRI approaches such as T_1_/T_2_ ratio or magnetisation transfer ratio (MTR) may also offer complementary contrast that may uncover lesions invisible on conventional inversion recovery sequences. These techniques have high sensitivity but thus far low specificity when it comes to alterations in the cortex [31, 35, 36]. Furthermore, artificial intelligence-based techniques may be utilised to boost sensitivity for cortical lesions and offer promising avenues for enhancing cortical lesion detection in a clinically feasible manner, [37, 38], but research is also focusing on, for example, cortical cellularity. [39]

A limitation to this work is that no 3D-PSIR images were available. As a result, multiplanar assessment of these images was not possible. Implementing a high-resolution 3D-PSIR acquisition with multiplanar reformatting is likely to improve lesion conspicuity and reduce partial volume effects, which have been observed in our current study. While deep grey matter involvement is indicated in MS, this region was not included due to the retrospective nature of the study and limited histological sampling of these regions [40]. Furthermore, no histopathological data on enlarged perivascular spaces were available. Therefore, we were not able to provide clear guidelines to discriminate between cortical lesions and juxta-/intracortical enlarged perivascular spaces.

In conclusion, our study highlights that the detection of cortical lesions in progressive multiple sclerosis remains challenging, even when employing advanced MRI sequences in a controlled postmortem setting. While combining DIR and PSIR sequences increased detection rates by 43% compared to single-sequence assessments, overall sensitivity remained low, with only 17.4% of histopathologically confirmed lesions identified prospectively. This underscores the persistent limitations of current MRI technology for cortical lesion detection, particularly for subpial lesions. Our findings also emphasise that the availability and combination of advanced MRI sequences have a significant impact on cortical lesion detection, as conventional clinical sequences alone detect only half as many lesions. Importantly, the use of consensus reading and strict lesion identification criteria ensured high specificity, with no false positives, but may have further limited sensitivity. Additionally, our results suggest that imaging plane orientation and the inherent limitations of 2D sequences contribute to missed lesions, indicating that future criteria should consider multiplanar assessment. Overall, cortical lesion detection remains limited in the current clinical setting with the combination of DIR and PSIR sequences providing the most effective MRI contrasts.
